# Uncovering the antiproliferative potential of *Lobophytum pauciflorum* metabolites through chemoinformatics and in vitro approaches

**DOI:** 10.1038/s41598-026-45881-8

**Published:** 2026-04-13

**Authors:** Aya Ali Alassass, Marwa S. Abu Bakr, Asmaa A. Mahmoud, Mona M. Eid, Seif-Eldin N. Ayyad, Abd El-Salam I. Mohammed

**Affiliations:** 1 Department of Pharmacognosy, Faculty of Pharmacy (for Girls), Al-Azhar University, Nasr City, Cairo, 11651 Egypt; 2 Department of Pharmacognosy, Faculty of Pharmacy, Horus University, Damietta, 34518 Egypt; 3 Department of Chemistry, Faculty of Science, Damietta University, Damietta, Egypt; 4 Department of Pharmacognosy, Faculty of Pharmacy (for Boys), Al-Azhar University, Nasr City, Cairo, 13129 Egypt

**Keywords:** *Lobophytum pauciflorum*, UHPLC-QTOF-MS/MS, Phytochemical profiling, Anticancer agents, Biochemistry, Biotechnology, Cancer, Chemical biology, Chemistry, Computational biology and bioinformatics, Drug discovery

## Abstract

Marine organisms such as soft corals represent a promising source of bioactive compounds for pharmaceutical development. However, studies on the antiproliferative activity of the Red Sea soft coral *Lobophytum pauciflorum* remain limited. This study aimed to investigate the chemical diversity of *L. pauciflorum* through a chemoinformatics approach combined with in vitro analyses. Chemical composition was characterized using ultra-high-performance liquid chromatography quadrupole time-of-flight tandem mass spectrometry (UHPLC-QTOF-MS/MS) for metabolite annotation. Anticancer activity was evaluated against eight human cancer cell lines, with the extract showing significant cytotoxicity against MDA-MB-231 and MCF-7 cells, exhibiting IC₅₀ values of 9.46 and 11.59 µg/mL, respectively. These findings indicate that *L. pauciflorum* possesses anticancer potential, and isolated compounds from its methanolic extract warrant further evaluation.

**Supplementary Information:**

The online version contains supplementary material available at 10.1038/s41598-026-45881-8.

## Introduction

Marine organisms are recognized as a major source of novel bioactive compounds, making the isolation and characterization of naturally occurring biologically active products a central focus of the global scientific community^1^. Among marine taxa, soft corals have emerged as particularly rich sources of secondary metabolites. Since the 1960s, when research on marine natural products was first initiated, soft corals have consistently yielded the highest number of newly reported metabolites compared with other marine plants and animals^2^.

More than 40 species of soft corals belonging to the genus *Lobophytum* (phylum Cnidaria, class Anthozoa, subclass Octocorallia, order Alcyonacea, family Alcyoniidae) are distributed across tropical and subtropical waters. Owing to the chemical diversity of marine ecosystems, these organisms represent an important source of secondary metabolites with diverse pharmacological activities. Members of the genus *Lobophytum* are abundant in coral reef habitats worldwide and are known to produce a wide array of structurally unique secondary metabolites with potential bioactivity^3–5^. Distinctive in their range of colors, morphologies, and sizes, *Lobophytum* species have yielded metabolites characterized by both strong biological activities and novel skeletal frameworks^6^.

Approximately 15 species of *Lobophytum* have been chemically investigated, resulting in the identification of more than 250 distinct compounds including cembranoids-type diterpenoids, sesquiterpenoids, and steroids^7,8^. These metabolites have attracted considerable interest from pharmacologists and chemists due to their wide range of bioactivities, including antimalarial, antibacterial, anticancer, and anti-inflammatory properties^9–15^.

The harsh environmental conditions of the arid Red Sea compel marine organisms to develop unique adaptive strategies for survival. However, to the best of our knowledge, investigations of soft corals from this region, particularly *Lobophytum pauciflorum*, remain limited.

Cancer arises from the accumulation of genetic abnormalities induced by various stimuli, including viral infections, ultraviolet radiation, and chemical carcinogens. According to the cancer immunoediting hypothesis, these abnormal cells (cancer cells) generated in the immunocompetent hosts are eliminated by the immune system (elimination phase)^16^. However, some cancer cells that can survive in the immunocompetent hosts by reducing the expression of highly immunogenic cancer antigens derived from gene alterations and/or MHC molecules are selected (equilibrium phase). In addition, cancer cells employ several immune-suppressive machineries, including inhibitory immune-checkpoint molecules and immunosuppressive cells such as regulatory T (Treg) cells, tumor-associated macrophages (TAMs), and myeloid-derived suppressor cells (MDSCs) to escape from antitumor immune responses (escape phase). Eventually, in immunocompetent hosts, they can grow and progress to become clinically apparent cancers^17,18^.

This study aimed to explore the chemical diversity of *L. pauciflorum* using a chemoinformatics approach combined with in vitro analyses. Metabolomic profiling of biomolecules was conducted using ultra-high-performance liquid chromatography quadrupole time-of-flight tandem mass spectrometry (UHPLC-QTOF-MS/MS). Furthermore, the cytotoxic activity of *L. pauciflorum* extract was evaluated against cancer cell lines. In addition, Chemoinformatic analyses were conducted to assess structural similarity and drug-likeness properties of the annotated metabolites.

## Methods

### Cell line

For screening, Hepatocellular carcinoma (HePG-2), Epitheliod carcinoma (HeLa), Epidermoid carcinoma (HEP2), Human prostate cancer (PC3), Mammary gland (MCF-7), Colorectal adenocarcinoma (Caco-2), Colorectal carcinoma (HCT-116), and Breast cancer (MDA-MB-231) The cell lines were originally obtained from the American Type Culture Collection (ATCC) and supplied locally through the Holding Company for Biological Products and Vaccines (Vacsera, Egypt). Fetal bovine serum was purchased from Gibco (UK). RPMI-1640 medium, 3-(4,5-dimethylthiazol-2-yl)−2,5-diphenyltetrazolium bromide (MTT), dimethyl sulfoxide (DMSO), and doxorubicin were purchased from Sigma Co. (USA).

### Soft coral materials and preparation of extracts

A soft coral of *Lobophytum pauciflorum* was collected from the Red Sea. A vacuum rotary evaporator was used to concentrate 20 g of dried soft coral sample after it had been macerated in 1:1 Methylene chloride to Methanol over three separate 24-hour periods. The yield extract was weighed and stored at − 20 °C.

### Sample preparation for UHPLC-QTOF-MS/MS analysis

UHPLC-QTOF-MS/MS analysis was performed according to a previously published article^19^. 50 mg of the lyophilized extract was diluted in 1 mL of the solvent combination made up of water, methanol, and acetonitrile (H_2_O: MeOH: ACN) in a (50: 25: 25) V/V ratio to create a stock solution of the extract. The sample was vortexed for 2 min and ultrasonically sonicated for 10 min to achieve complete solubility of the stock solution. After centrifuging the stock solution at 10,000 rpm for 10 min. 50 µL of stock solution was diluted to 1000 µL with H_2_O: MeOH: ACN (50: 25: 25) V/V, and finally, the injected concentration was 2.5 µg/µL. A 10 µL sample of injection was used for both positive and negative modes. Moreover, 10 µL of reconstitution solvent was injected as a blank sample^20,21^.

### UHPLC-QTOF-MS/MS acquisition parameters

Solution (A) was the positive mode mobile phase, comprising 5 mM ammonium formate in 1% methanol, pH adjusted with formic acid to 3. Solution (B) was used as the negative mode mobile phase and consisted of 5 mM ammonium formate in 1% methanol with the pH adjusted to approximately 8.0 using sodium hydroxide. Solution (C) comprised 100% acetonitrile for both the negative and positive modes. With a flow rate of 0.3 mL/min, the gradient elution was carried out using the following programs: for positive mode: (1) 0–20 min, 95% for A and B − 5% for C; 21–28 min, 5% for A and B − 95% for C; and 28.1–35 min, 95% for A and B − 5% for C, (2) for both negative and positive modes. Compounds were separated using an X select HSS T3 (2.5 μm, 2.1 mm × 150 mm) column (Waters, USA) and in-line filter disks pre-column (0.5 μm × 3.0 mm, Phenomenex, USA) conditioned at 40 °C. Chromatographic separation was performed using an Exion LC™ Series UHPLC system. Mass spectrometric detection was carried out using a TripleTOF 5600 + mass spectrometer (Sciex, USA) equipped with an electrospray ionization (ESI) source operating in both positive and negative ion modes. The information-dependent acquisition (IDA) method was used, and instrument control was performed using Analyst-TF 1.7.1 software^22^.

### Data processing and feature extraction

Utilizing the open-source MS-DIAL 4.9 tool, the material was comprehensively analysed using small molecules and non-targeting techniques. GNPS negative (2,351 records), and GNPS positive (8,782 records) databases were used as reference databases. The MasterView was used for feature (peaks) extraction from Total ion chromatogram (TIC) based on the following criteria; 1- Features should have Signal-to-Noise greater than 10 (non-targeted analysis). Features recommended for achieving a ppm error of less than or equal ± 5 (non-targeted analysis).

The MS dereplication workflow involved feature detection, peak alignment, and annotation using the MS-DIAL platform. Detected features were first filtered based on signal-to-noise ratios and accurate mass tolerance (≤ 5 ppm). The resulting molecular features were then matched against public spectral libraries including GNPS and PubChem. Tentative metabolite annotation was achieved through comparison of accurate mass values, MS/MS fragmentation patterns, and literature data. In cases where reference spectra were unavailable, structural assignment was supported by characteristic fragmentation pathways of the corresponding chemical classes.

### Chemoinformatic analysis through Tanimoto similarity

Chemoinformatic analysis was used to determine the structural links between the annotated metabolites discovered in the extract^23^. The Tanimoto similarity coefficient, computed from binary chemical fingerprints, was used to analyze pairwise structural similarities. The simplified molecular input line-entry system (SMILES) for each molecule was obtained from PubChem (https://pubchem.ncbi.nlm.nih.gov/). Computations were carried out in Python 3.10.16 with the RDKit package^24^. Morgan fingerprints (radius = 2, 2048 bits) were utilized to indicate the presence or absence of molecular substructures. The resultant fingerprints were analyzed using the Tanimoto coefficient to get a sense of structural similarity and scaffold diversity among the identified metabolites. This approach was designed to depict the chemical space represented by the metabolite collection, rather than to infer ionization behavior or analytical preference. Tanimoto similarity was then calculated as:

$$\:T(A,B)=\frac{c}{a+b-c}$$

In this calculation, *a* and *b* represent the number of “presence” bits in compounds *A* and *B*, respectively, while *c* denotes the number of shared “presence” bits between them. Tanimoto similarity values above 0.7 indicate high structural similarity, values between 0.4 and 0.7 indicate moderate similarity, and values below 0.4 suggest substantial scaffold diversity^23^.

### MTT assay

Before being used, the extract was sterilized by passing through a Millipore filter (0.22 μm) and diluted with cell culture medium to different working concentrations of 100, 50, 25, 12.5, 6.25, 3.125, and 1.56 µg/mL. The extract was prepared in DMSO (Sigma Aldrich) at a concentration of 1000 µg/mL. The MTT technique was used to assess the extract ability to impede the growth of the four human tumor cell lines. This colorimetric method relies on mitochondrial succinate dehydrogenase in live cells, turning the yellow MTT into a purple formazan derivative. The cells were purified using 10% fetal bovine serum in RPMI-1640 media. At 37 °C in an incubator with 5% CO_2_, doxorubicin was introduced as antibiotics. In a 96-well plate, the cells were seeded at a density of 1.0 × 10^4^ cells per well and kept at 37 °C for 48 h with 5% CO_2_. The cells were cultured for 24 h after being treated with different concentrations of the extract. Following a 24-hour treatment period, 20 µL of MTT solution (5 mg. mL^− 1^) was added, and the mixture was incubated for four hours. Each well received 100 µL of DMSO to dissolve the purple formazan that had developed. A plate reader (EXL 800, USA) was used to quantify the colorimetric assay’s absorbance at 570 nm. The following formula was used to determine the relative cell viability as a percentage: [(A570 of treated sample)/(A570 of untreated sample)] × 100 ^25^. All experiments were performed in three independent experiments, each conducted in triplicate. IC₅₀ values were calculated from dose–response curves using nonlinear regression analysis and are expressed as mean ± standard deviation (SD).

### Drug-likeness analysis

Drug-likeness analysis was performed using SwissADME (http://www.swissadme.ch/index.php)^26^. The evaluation included Lipinski, Ghose, Veber, Egan, and Muegge rules, as well as medicinal chemistry filters such as pan-assay interference compounds (PAINS), Brenk alerts, and lead-likeness.

## Results

### Antiproliferative activity of *L. pauciflorum* methanolic extract

**Fig. 1:**
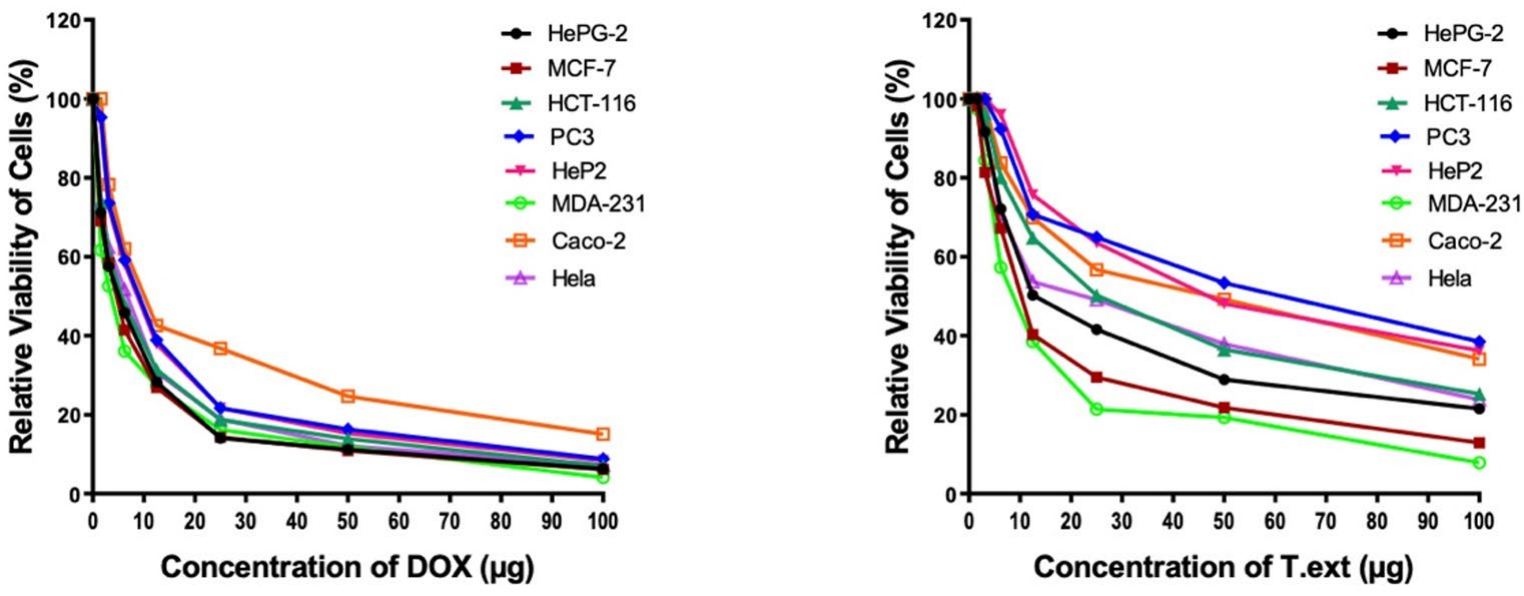
Effect of doxorubicin and *L. pauciflorum* extract on the relative viability of various cancer cell lines.

The antiproliferative activity of *L. pauciflorum* extract (T.ext) was evaluated against eight human tumor cell lines and compared with the reference chemotherapeutic agent doxorubicin (DOX). Both DOX and T.ext exhibited dose-dependent cytotoxic effects across the tested cell lines. DOX demonstrated strong cytotoxic activity across all investigated cell lines, whereas T.ext showed moderate to weak activity against several cell lines but strong activity against MCF-7 and HepG-2 and significant activity against MDA-MB-231. At 100 µg/mL, DOX reduced cell viability to less than 10% in most cell lines, whereas T.ext produced varying degrees of cytotoxicity, with the most pronounced effects observed in MDA-MB-231 and MCF-7 cells (Tables 1, 2; Fig. 1).

**Table 1:** Cytotoxic activity of some compounds against human tumor cells.

Comp.	In vitro cytotoxicity IC_50_ (µg/ml)							
	HePG-2	MCF-7	HCT-116	PC3	HeP2	MDA-231	Caco-2	Hela
DOX*	4.50 ± 0.2	4.17 ± 0.2	5.23 ± 0.3	8.87 ± 0.6	8.54 ± 0.6	3.18 ± 0.1	12.49 ± 1.1	5.57 ± 0.4
T.ext	18.11 ± 1.3	11.59 ± 0.9	27.65 ± 1.8	54.35 ± 3.2	48.82 ± 2.8	9.46 ± 0.7	42.21 ± 2.4	23.06 ± 1.5

Cytotoxic activity classification follows established criteria based on IC₅₀ thresholds: potent (< 1 µM or < 0.5 µg/mL), notable (1–10 µM or 0.5–5 µg/mL), moderate (10–30 µM or 5–15 µg/mL), mild (30–50 µM or 15–25 µg/mL), and negligible (> 50 µM or > 25 µg/mL) as outlined by Zhang et al.^27^. DOX*: Doxorbicin.

**Table 2:** Average of relative viability of cells (%).

Conc.(µg)	HePG-2	MCF-7	HCT-116	PC3	HeP2	MDA-231	Caco-2	Hela
*DOX*								
100	6.3	6.2	7.1	8.8	8.4	4.1	15.1	7.3
50	11.2	10.9	13.9	16.3	15.3	11.8	24.7	12.1
25	14.1	14.3	18.7	21.7	21.7	16.2	36.8	18.9
12.5	28.3	26.9	31.4	38.9	37.9	27.3	42.6	30.8
6.25	45.8	41.5	47.9	59.2	58.2	36.1	62.0	51.7
3.125	57.6	58.4	60.5	73.6	72.6	52.6	78.3	62.4
1.56	71.2	69.1	73.8	95.3	94.3	61.7	100	74.0
*T.ext*								
100	21.5	12.9	25.3	38.5	36.2	7.9	34.1	23.8
50	28.9	21.8	36.5	53.4	48.1	19.3	49.2	37.9
25	41.6	29.5	50.2	64.9	63.5	21.4	56.7	49.1
12.5	50.3	40.3	64.8	70.7	75.7	38.5	69.9	53.7
6.25	72.1	67.2	80.1	92.3	96.0	57.3	83.8	70.6
3.125	91.7	81.4	96.3	100	100	84.4	98.5	92.3
1.56	100	98.1	100	100	100	97.4	100	100

### UHPLC-QTOF-MS/MS screening of secondary metabolites from the soft coral *L. pauciflorum*

The identification of the metabolites is mainly based on the interpretation of their SIC and MS2- spectra using a peak-view software version (Peak view 1.2) (AB SCIEX, US) with the four-digit high-resolution *m*/*z* values. The analyses were run in both ion modes to afford sufficient, diagnostic, and structurally relevant fragments that aid in the structure identification for 24 metabolites in both modes, including phenolic acid with its derivatives and carboxylic acids, triterpene, diterpenes, sesquiterpenes, fatty acid, alkaloids, polyketide, and alcohol. The fragmentation patterns of the compounds were predicted according to previously reported in the literature in addition to open-access mass-spectra databases such as PubChem. Moreover, the peaks were numbered according to their elution order along with their metabolite names, molecular formula, and the nature of the metabolite, as well as their experimental m/z, error calculations in ppm, and MS/MS fragments. Furthermore, the high‑resolution mass error values are calculated using the following formula: ∆*mi* = (*mi* – *ma*)/*ma* ×10^6^, where ∆*mi* = mass errors, *mi* = measured mass, and *ma* = exact mass^28^, where metabolites with a high-resolution mass error of more than 5 ppm were excluded^29^. The detailed metabolite profiles are summarized in Tables 1 and 2; and are further illustrated in Fig. 1 and in the supplementary Figures S1 and S2. The compound numbers shown in Tables 3 and 4 were used as the reference identifiers for all subsequent chemoinformatic analyses.

**Fig. 2:**
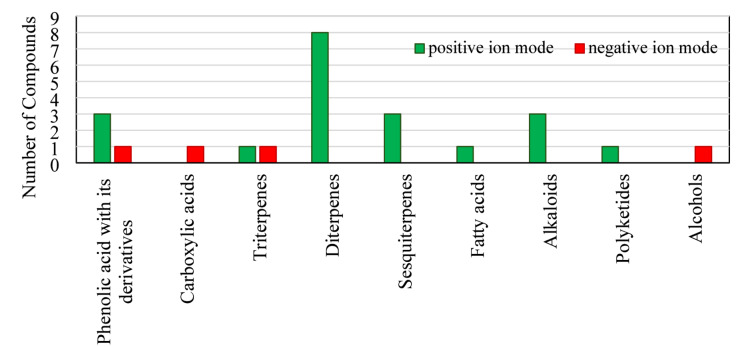
Comparison of the number of compounds identified under each chemical class in positive and negative ion modes of soft coral *Lobophytum pauciflorum* by UHPLC-QTOF-MS/MS.

According to metabolomics reporting standards, the identified metabolites were classified as Level 2 annotations (putatively annotated compounds) based on accurate mass measurements, MS/MS fragmentation patterns, and comparison with public spectral databases and literature reports (Fig. 2).

**Table 3:** Phytochemical profiling of soft coral *L. pauciflorum* by UHPLC-QTOF-MS/MS in negative ion mode.

Compound no.	Metabolite name	Molecular formula	Rt	Error (PPM)	Experimental mass [M-H]^−^	Chemical class	Fragmentation (m/z)	Refs.
1.	Gallic acid	C_7_H_6_O_5_	7.88	1.2	169.0135	Phenolic acid	169.01, 151.07, 132.92, 125.09, 107.09, 97.06, 79.05, 59.01	^30,31^
2.	Maleic acid	C_4_H_4_O_4_	10.59	− 0.9	115.0030	Carboxylic acid	115.00, 71.01	^32^
3.	Benzyl alcohol	C_7_H_8_O	16.61	− 3.7	107.0492	Aromatic alcohol	107.04, 105.07, 92.05, 91.05, 81.03, 79.05, 77.08, 66.04, 65.03, 63.02, 55.01, 53.03, 51.02	^33^
4.	Maslinic acid	C_30_H_48_O_4_	21.70	− 0.8	471.3470	Triterpene	471.34, 423.03, 405.09, 393.09	^34–37^

**Table 4:** Phytochemical profiling of soft coral *L. pauciflorum* by UHPLC-QTOF-MS/MS in positive ion mode.

Compound no.	Metabolite name	Molecular formula	Rt	Error (PPM)	Experimental mass[M + H]^+^	Chemical class	Fragmentation	Refs.
5.	4-Hydroxy-3,6-dimethylpyran-2-one	C_7_H_8_O_3_	1.25	4.3	141.0557	Polyketide	141.05, 123.05, 105.07, 97.07, 95.09, 56.04	^38^
6.	Trigonelline	C_7_H_7_NO_2_	1.36	1.4	138.0558	Alkaloids	138.05,123.01, 107.07, 94.06, 92.04,78.03	^36^
7.	4,6-Dimethoxyfuro[2,3-b]quinolone (Pteleine)	C_13_H_11_NO_3_	6.51	− 0.9	230.0815		230.08, 215.05, 200.10, 186.12	^37^
8.	*trans*-Cinnamaldehyde	C_9_H_8_O	11.22	0.00	133.0653	Phenolic	133.06, 118.07, 115.05, 105.06, 103.05, 91.05, 78.76, 65.03, 55.06	^39^
9.	14-Deoxy-11,12-didehydroandrographolide	C_22_H_26_O_7_	13.38	− 0.9	333.2062	Diterpene	333.20, 315.05, 303.08, 296. 91, 285.09, 268.87, 257.08, 240.87, 240.12, 218.89, 205.01, 200.88, 174.89	^40^
10.	Isopetasol	C_15_H_22_O_2_	13.43	0.00	235.1698	Sesquiterpene	235.16, 217.15, 199.15, 198.94,189.16, 175.15, 159.11,149.07, 135.05, 131.08, 123.09, 109.07, 105.07, 95.08,84.95,81.02	^41^
11.	Saurufuran B	C_20_H_28_O_3_	15.21	0.00	317.2116	Furanditerpene	317.21, 316.92, 299.23, 270.90, 252.89, 203.11, 185.09, 151.10, 133.06	^42^
12.	Gallic acid	C_7_H_6_O_5_	15.28	− 1.8	171.0290	Phenolic acid	171.02, 153.04, 135.06, 127.07, 125.03, 112.10, 109.04, 89.07, 88.95, 84.96, 81.06, 72.04, 70.97, 69.07, 53.08	^43^
13.	Cafestol	C_20_H_28_O_3_	15.87	− 1.6	317.2111	Diterpene	317.21, 299.19, 298.90, 280.89, 270.90, 255.21, 253.19, 252.89, 243.14, 224.89, 217.12, 199.14, 197.13, 187.07, 171.11	^44^
14.	Alismoxide	C_15_H_26_O_2_	16.00	− 0.4	239.2010	Sesquiterpene	239.20, 203.17, 161.14, 147.11, 119.08, 105.09, 95.04	^45^
15.	Kaurenic acid	C_20_H_30_O_2_	16.35	− 0.3	303.2325	Diterpene	303.23, 285.23, 243.19, 238.87, 216.88, 183.11, 159.11, 131.08, 117.07, 91.05, 90.97	^46,47^
16.	Harmine	C_13_H_12_N_2_O	16.84	− 0.9	213.1027	Alkaloids	213.10, 198.10, 197.09, 170.10,154.07, 143.08, 141.07, 128.06, 115.05	^48^
17.	*ar*-Abietatriene	C_20_H_30_	16.88	0.00	271.2425	Diterpene	271.24, 255.04, 229.19, 206.90, 187.14, 173.12, 159.12, 149.12, 119.08, 105.07	^42,46^
18.	7-Oxydehydroabietic acid	C_20_H_26_O_3_	17.25	− 3.2	315.1950	Diterpene	315.19; 297.03, 278.02, 269.18, 241.16, 199.01,187.11, 171.07	^49,50^
19.	15-Hydroxy-7-*oxo* dehydroabietic Acid	C_20_H_26_O_4_	17.52	− 1.8	331.19030	Diterpene	331.19, 313.22, 281.18, 254.88, 248.92, 230.91, 215.03, **203.02**	^50^
20.	Arachidonic acid	C_20_H_32_O_2_	18.62	0.00	305.2480	Fatty acid	305.24, 286.91, 269.14, 259.12, 240.90, 208.93, 190.92, 151.07, 149.09, 137.05, 109.06, 79.05, 81.07	^42,51^
21.	Maslinic acid	C_30_H_48_O_4_	19.93	0.2	473.3631	Triterpene	473.36, 455.35, 437.35, 409.06	^35^
22.	Prespatane	C_15_H_24_	20.42	0.5	205.1957	Sesquiterpene	205.19, 189.89, 177.12, 159.11, 149.13, 135.12, 123.08, 121.10, 110. 02, 107.08, 95.08, 93.07, 81.02	^52^
23.	Totarol	C_20_H_30_O	20.47	− 3.5	287.2364	Diterpene	287.23, 269.22, 241.09, 227.07, 223.08, 218.06, 200.92, 173.12, 147.11, 133.10, 119.08, 97.10, 91.05	^53^
24.	Dimethoxy-4-hydroxybenzaldehyde (Syringaldehyde)	C_9_H_10_O_4_	22.81	− 1.1	183.0654	Phenolic compounds	183.06, 168.06,165.13, 140.87, 138.12, 123.94	^54^

### Chemoinformatic similarity analysis

**Fig. 3:**
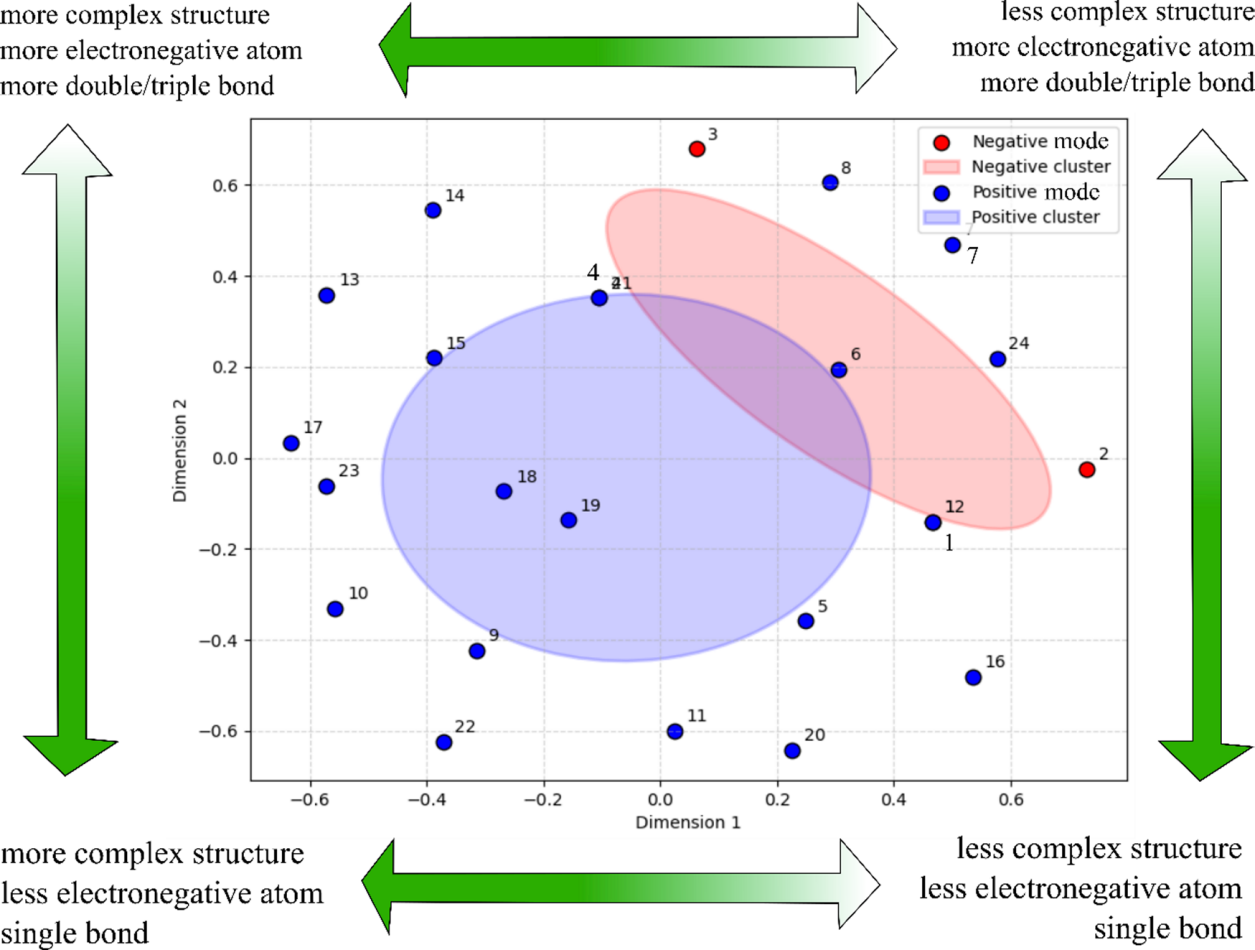
Tanimoto similarity based on binary molecular fingerprint.

In the chemoinformatic analysis, Tanimoto similarity was applied to molecular fingerprints. Molecular fingerprints reflect the presence or absence of specific substructures within a compound, whereas molecular descriptors capture its physicochemical properties. Compounds detected in negative ionization mode were predominantly enriched in acidic or highly polar functional groups, resulting in moderate molecular weights (300–400 g/mol) and elevated TPSA values (> 100 Å²). In contrast, compounds identified in positive ionization mode occupied a wider physicochemical space, exhibiting greater variability in both molecular weight and TPSA (Fig. 3).

### Drug-likeness analysis

**Fig. 4:**
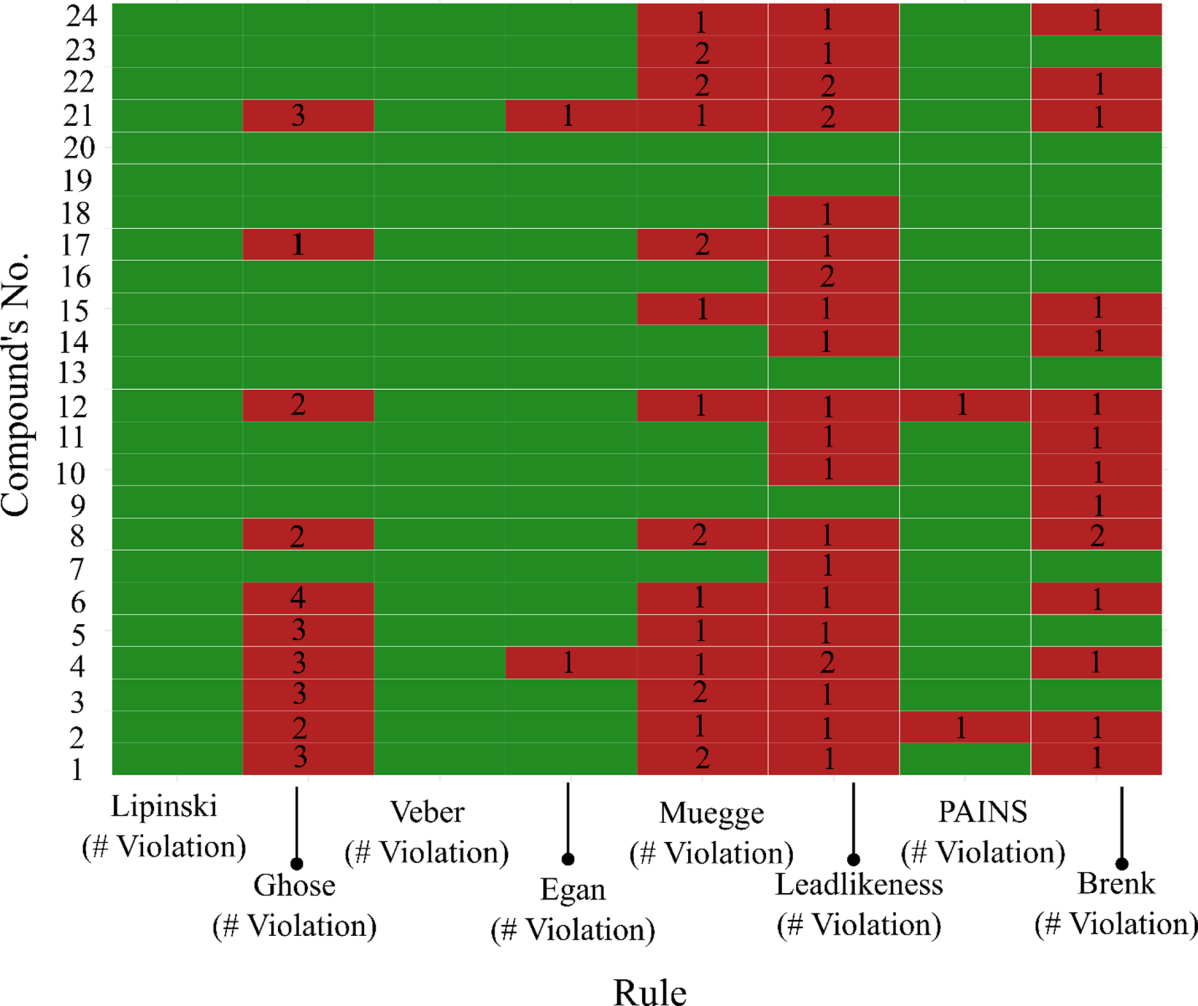
The ADME analysis of the identified compound. The number inside the box represents the number of violations. The green color showed a compliment to the rules, while the red color showed no such compliment.

Drug-likeness of the identified compounds was evaluated using SwissADME. All compounds complied with Lipinski’s rule of five and Veber’s rule, although some exhibited a single violation for Lipinski’s rule. Ten compounds (41.7%) violated the Ghose rule and two violated the Egan rule (Fig. 4). In contrast, most compounds did not satisfy the Muegge rule, with only eight (33%) meeting its criteria. More compounds passed the PAINS rule regarding medicinal chemistry filters compared with the Brenk and lead-likeness filters. Compounds 9, 13, 19, and 20 showed no violation to the Lipinski, Veber, Ghose, Egan, and Muegge rules.

## Discussion

Phenolic acids and their derivatives are characterized by decarboxylation [M-H-CO_2_] ^−^ and dehydration [M-H-H_2_O]^31,55^. These fragmentations occurred in gallic acid structure, which showed at *m/z* 169.0135 [M-H] ^−^ (peak 1), and at *m/z* 171.0290 [M + H]^+^ (peak 8)with its major fragment ions at *m/z* 151.07 and 153.04 [M-H± H_2_O] ^±^ and 125.09, and 127.07 [M ± H- CO_2_]^±^ in the negative and positive modes, respectively^30,43^. Moreover, in the positive mode, peak 4 appeared at *m/z* 133.0653 [M + H]^+^ with its fragment ion at *m/z* 115.05 [M + H− H_2_O]^+^, and other fragments are shown in Table 2, which are characteristic of *trans*-cinnamaldehyde^39^. Also, peak 20 was assigned at *m/z* 183.0654 [M + H]^+^ and fragment ions *m/z* at 168.06 [M + H− CH_3_]^+^, 165.13 [M + H−H_2_O]^+^ which corresponding to Dimethoxy-4-hydroxybenzaldehyde (Syringaldehyde)^54^. On the other hand, one carboxylic acid (non-phenolic) in the negative mode peak 3 showed at the deprotonated molecule ion [M-H] ^−^ at *m/z* 115.0030 and its fragment at *m/z* 71.01 [M-H-CO_2_] ^−^ corresponding to Maleic acid^32^.

Diterpenes are characterized by multiple fragmentation patterns in mass spectrometry analysis as, [M − H − CH_3_]^−^, [M − H − H_2_O]^−^, [M − H − CO_2_]^−^ and [M − H − H_2_O − CO_2_]^−^, [M − H − H_2_O − CO]^−^and [M − H − H_2_O − CO − CH_3_]^−^, [M + H-HCHO]^+^ and retro Diels –Alder (RDA) cleavage mechanism^56,57^. also, cleavage of Alkyl side chains(isopropyl groups)^58^. A dominant fragmentation pathway involves the initial elimination of a water molecule. This dehydration can occur either via direct protonation on an oxygen atom or through a rearrangement involving hydrogen migration. The resulting formation of an endocyclic double bond often facilitates a subsequent retro-Diels-Alder (RDA) cleavage, which is identified as a primary fragmentation mechanism for ring systems^59^. In the positive mode, peak 5, 7, 9, 11, 14, 15, and 19 were appeared at *m/z* 333.2062, 317.2116, 317.2111, 303.2325, 315.1950, 331.19030, and 287.2364 [M + H]^+^ with most common fragment ions among them at *m/z* 315.05, 299.23, 299.19, 285.22, 297.03, 313.22 and 269.22 [M + H − H_2_O]^+^ and other fragments are summarized in Table 2 according to the literature which corresponds to 14-Deoxy-11,12-didehydroandrographolide, Saurufuran B, cafestol, kaurenic acid, 7-oxydehydroabietic acid, 15-hydroxy-7-*oxo* dehydroabietic Acid and Totarol, respectively^40,42,44,46,47,49,50^. Also, peak 13 was showed at protonated ion at *m/*z 271.2425 [M + H]^+^ with their fragments at *m/*z 255.05 [M + H- CH_4_]^+^, 229.19 [M + H- C3H_7_]^+^ loss of isopropyl groups, and fragment ion at *m/z* 173.12. By comparing these fragmentation spectra with literature reports for metabolites with the same chemical class that corresponds to ar-abietatriene^42^.

Sesquiterpenes undergo multiple fragmentation patterns in mass spectrometry such as [M + H-CH_3_]^+^, [M + H- C3H_6_]^+,^ [M + H − H_2_O]^+^, [M + H-H_2_O-CH_3_]^+^, [M + H−CO_2_]+, [M + H-H_2_O-C_3_H_6_]^+^, [M + H*−*H_2_O *−* CO]^+^, [M + H-C_2_H_4_]^+^, [M + H- C_4_H_8_]^[+ 60,61^. In the positive mode, these fragmentations were observed clearly in peaks 8, 12, and 17. Peak 6 was showed at protonated ion at *m/*z 235.1698 [M + H]^+^ and product ions at *m/z* 217.15 [M + H- H_2_O]^+^, 199.15 [M + H-2H_2_O]^+^, 189.16 [M + H *−* H_2_O *−* CO]^+^, 175.15 [M + H-H_2_O-C_3_H_6_]^+^ loss of water followed by isopropyl group, and other fragments as 149.07, 123.09, 109.07, 95.08, and 81.02 are major product ions in bicyclic sesquiterpenes. By comparing their fragmentation spectra with database and literature reports for comparable metabolites with the same chemical class^41,62^, this metabolite was attributed to isopetasol. Peak 10 was observed at *m/z* 239.20 [M + H]^+^ and product ions 203.17[M + H-_2_H_2_O]^+^, 161.14 [M + H- 2H_2_O-C_3_H_6_]^+,^ loss of 2 H_2_O molecule followed by isopropyl group, 147.09 [M + H- C_4_H_8_]^+^, 119.08, 105.09, 95.06, which corresponds to Alismoxide^45^. Peak 17 was shown at m/z 205.1957 [M + H]+ with daughter ions at m/z 189.89 [M + H-CH3]+, corresponding to Prespatane^52^.

In the positive mode, three alkaloids were identified. The first one was trigonelline (peak 2), which appeared at *m/z* 138.0558 [M + H]^+^ with major ion peaks at *m/z* 123.01 [M + H- CH_3_]^+^_,_ 107 [M + H- OCH_3_]^+^, and 94[M + H-CO_2_]^[+ 36^. Also, Peak 3 was showed at *m/z* 230.081 [M + H]^+^ and product ions at *m/z* 215.05 [M + H- CH_3_]^+^, 200.10[M + H- CH_2_O]^+^, 186.06[M + H- CH_3_- CO]^+^. Metabolite was identified by comparing its fragmentation spectra with the database and literature reports for similar metabolites with the same chemical class^37,63^, this metabolite was attributed to Pteleine. Peak 12 displayed at *m/z* 213.1027 [M + H]^+^ with fragment ions at *m/z* 198.10 [M + H- CH_3_]^+^, 183.02 [M + H- OCH_3_]^+^, 170.01[M + H- CH_3_-CO]^+^ which corresponding to harmine^48^.

In the negative mode, peak 4 represents one aromatic compound, which was assigned to be deprotonated at *m/z* 107.0492 [M-H] ^–^, and its major fragments 79.05 [M + H- CO_2_]^−^ and other fragments are mentioned in Table 1, which corresponds to benzyl alcohol^33^. In the positive mode, Peak 1 belongs to polyketides (pyran derivative), was shown at *m/z* 141.0557 [M + H]^+^ and fragments at *m/z* 123.05[M + H-H_2_O]^+^, 105.07 [M + H-2H_2_O]^+^, 97.07[M + H-CO_2_]^+^, 95.09 [M + H-H_2_O-CO]^+^. By comparing these fragments with the database fragments^38^ and literature reports for similar metabolites^64,65^, this metabolite was attributed to 4-hydroxy-3,6-dimethylpyran-2-one. In addition, one fatty acid peak 17 was detected at the pseudo molecular ion peak at *m/z* 305.2480 [M + H]^+^, with its major daughter ions at *m/z* 286.91[M + H-H_2_O]^+^ indicating the presence of a hydroxyl group and at *m/z* 259.12 [M + H-H_2_O-CO]^+,^ which corresponds to Arachidonic acid^42,51^. Moreover, the presence of one Triterpene metabolite was observed in the negative mode (peak 4) at *m/z* 471. 3470 [M-H] ^–^ and major fragments at *m/z* 423.03 [M + H- 48]^−^, indicating loss of HCHO coupled with a H_2_O group, *m/z* 405.09[M + H–HCHO − 2H_2_O]^−^, indicating loss of 66 Da, *m/z* 393.09 due to loss of (78 Da), which is characteristic for maslinic acid^34,35^. In addition, in the positive mode, maslinic acid appeared (peak 17) at *m/z* 473.3631[M + H]^+^ 455.35[M + H-H_2_O]^+^, 437.35 [M + H-2H_2_O]^+^, 409.06 [M + H- CO − 2H_2_O]^[+ 35^.

Tanimoto similarity analysis was used to illustrate the structural links between annotated metabolites and offer an overview of scaffold diversity within the discovered metabolite pool. This chemoinformatic technique identifies clusters of structurally similar molecules as well as chemically different frameworks in the extract. It should be mentioned that neither the explanation of ionization efficiency nor the rationale for favoring a specific ionization method was the goal of the similarity analysis. Functional groups, polarity, and proton affinity are examples of physicochemical characteristics that largely control the differences between positive and negative ionization modes^35^. The greater number and broader chemical classes of metabolites found under these analytical circumstances are primarily responsible for the wider structural diversity seen in the positive mode of the current dataset. In addition, drug-likeness assessment was employed as an early-stage filtering strategy to evaluate essential physicochemical properties related to absorption, permeability, and molecular developability. Although these descriptors do not directly predict biological activity or mechanism of action, they provide useful guidance for selecting compounds that combine structural diversity with favorable pharmacokinetic properties^26^.

In this study, the cytotoxic effects of Doxorubicin (DOX) and *L. pauciflorum* extract on eight human cancer cell lines show an apparent dose-dependent decline in cell viability across all cell lines, indicating the expected cytotoxic effect of DOX. HePG-2 and MCF-7 cells exhibit the most extraordinary sensitivity, with significant reductions in viability at low concentrations. In contrast, cell lines such as MDA-MB-231, Caco-2, and HeLa display comparatively reduced sensitivity, maintaining higher viability percentages at equivalent DOX concentrations. The cytotoxic activity of *L. pauciflorum* extract demonstrates notable activity against several cell lines, with HePG-2, MCF-7, and HCT-116 emerging as the most responsive. The relative flattening of the curves in cell lines such as Caco-2 and HeLa suggests a lower susceptibility to the extract, potentially requiring higher concentrations for therapeutic efficacy. Cancer cells arise through the accumulation of genetic abnormalities caused by various stimuli, and the need for new, innovative, safe, and effective anticancer medications has increased due to the rising incidence of cancer-induced death worldwide. The study compares the cytotoxic effects of DOX and *L. pauciflorum* soft coral against eight human tumor cell lines.

The results of the study present a comparative analysis of the cytotoxic effects of two treatments, DOX (Doxorubicin) and *L. pauciflorum* soft coral against Eight human tumor cell lines namely; Hepatocellular carcinoma (HePG-2), Epitheliod Carcinoma (Hela), Epidermoid Carcinoma (HEP2), Human prostate cancer (PC3), Mammary gland (MCF-7), Colorectal adenocarcinoma (Caco-2), Colorectal carcinoma (HCT-116), and Breast cancer (MDA-MB-231). The relative viability of cells was measured across a range of concentrations (from 100 µg to 1.56 µg), providing insight into the dose-dependent response of each cell line to both treatments.

The *L. pauciflorum* soft coral extract exhibited dose-dependent cytotoxicity with selective antiproliferative activity across the tested cell lines. It showed strong activity against MDA-MB-231 and MCF-7 cells (IC₅₀ = 9.46 and 11.59 µg/mL, respectively), moderate activity against HeLa, HCT-116, Caco-2, and HeP2, and weak activity against PC3.

These results suggest that *L. pauciflorum* soft coral extract may have selective cytotoxicity, as it is more effective against certain tumor types while less effective against others. In addition, the limited activity against PC3 cells suggests a possible resistance or lack of specific molecular targets required for the extract’s action in this cell type. The antioxidant and cytotoxic activities of the organism and its capacity to biosynthesize patterns of phenolics as part of the potential mechanism to overcome oxidative stress are reflected in the fact that the majority of the identified compounds were phenolics. Phenolic compounds are widely reported to exhibit antioxidant and cytotoxic effects against various cancer cell lines through mechanisms including apoptosis induction, cell cycle arrest, and modulation of oxidative stress pathways^66,67^. These properties may contribute to the antiproliferative effects observed for the extract in the present study.

Our research indicates that cell survival decreased dose-dependently during the incubation period with doxorubicin Doxorubicin treatment resulted in a marked dose-dependent reduction in cell viability across all tested cell lines. At the highest tested concentration (100 µg/mL), viability of MDA-MB-231 cells decreased to approximately 4%, indicating strong cytotoxic activity. In contrast, *L. pauciflorum* extract also reduced cell viability in a dose-dependent manner but generally resulted in higher residual viability at equivalent concentrations, reflecting comparatively lower cytotoxic potency. These findings confirm the stronger overall cytotoxic effect of doxorubicin while indicating selective antiproliferative activity of the soft coral extract toward specific cell lines.

## Conclusion

The methanolic extract of *L. pauciflorum* revealed a diverse array of compounds. The presence of electronegative atoms and multiple bonds influenced their physicochemical characteristics, including variation in topological polar surface area. The extract exhibited dose-dependent antiproliferative activity against several cancer cell lines, showing the strongest effect against MDA-MB-231 with an IC_50_ of 9.46 ± 0.7 µg/mL. Further bioassay-guided fractionation and characterization of active constituents are required to confirm their therapeutic potential and to investigate possible synergistic effects with established chemotherapeutic agents. Positive ion mode demonstrates superior detection efficiency for the majority of compound classes, such as diterpenes, sesquiterpenes, fatty acids, triterpenes, polyketide, alkaloids, phenolic acid, and their derivatives; otherwise, negative mode represents phenolic acids, carboxylic acids, alcohol, and one triterpene.

## Supplementary Information

Below is the link to the electronic supplementary material.

Supplementary Material 1

## Data Availability

The data used in the study were presented in the manuscript.
