# Supplementary material for: Uncovering the antiproliferative potential of Lobophytum pauciflorum metabolites through chemoinformatics and in vitro approaches
Source: Sci Rep. 2026 Apr 13;16:12882. doi: 10.1038/s41598-026-45881-8 (PMC13096509; doi:10.1038/s41598-026-45881-8)
Supplement: Supplementary file 1 — Supplementary Material 1 [file 41598_2026_45881_MOESM1_ESM.docx]

**Supplementary data**

**Uncovering the antiproliferative potential of *Lobophytum pauciflorum* metabolites through chemoinformatics and *in vitro* approaches**

| 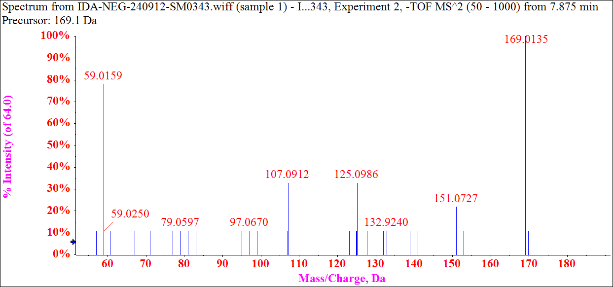 | 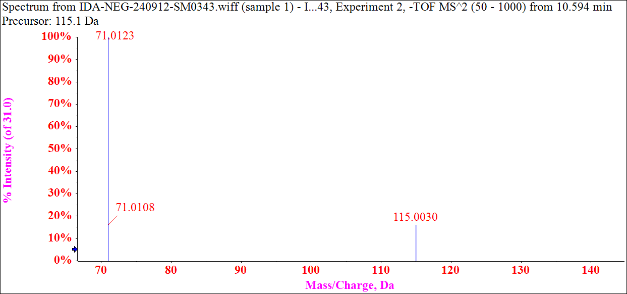 |
| --- | --- |
| 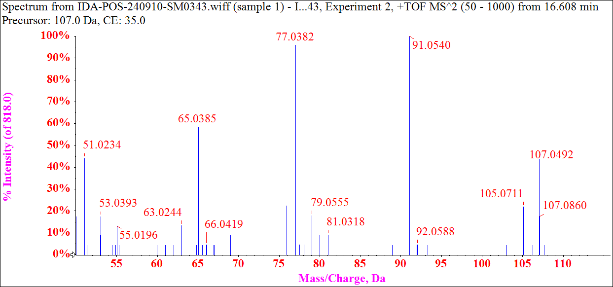 |  |

**Figure S1**. Fragmentation figures of the metabolites identified tentatively in soft coral *L. pauciflorum* by UHPLC-QTOF-MS/MS (positive mode).

| 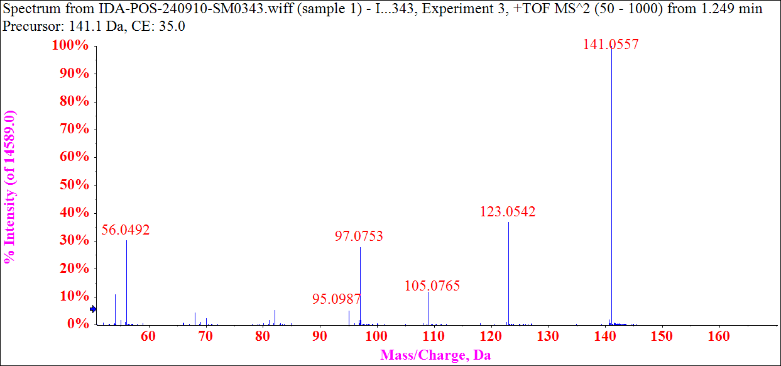 | 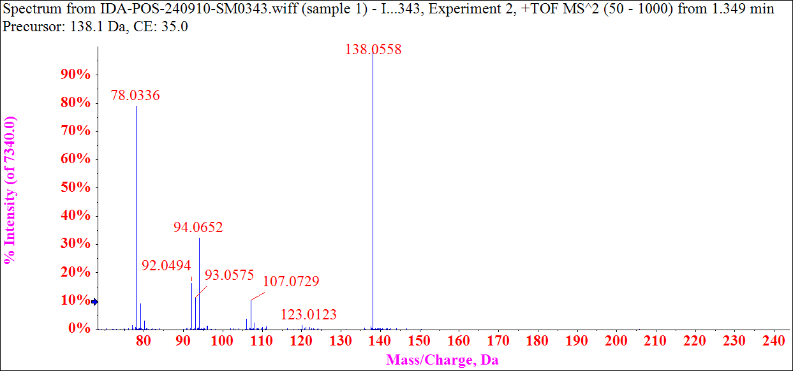 |
| --- | --- |
| 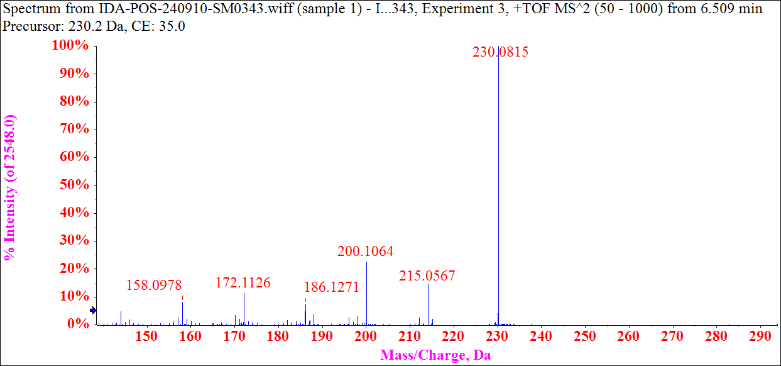 | 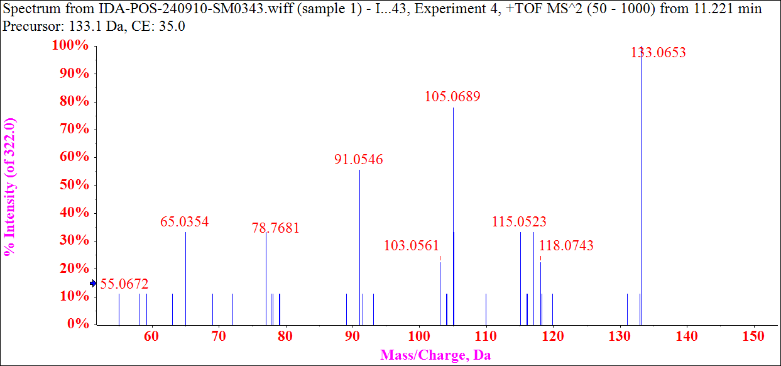 |

| 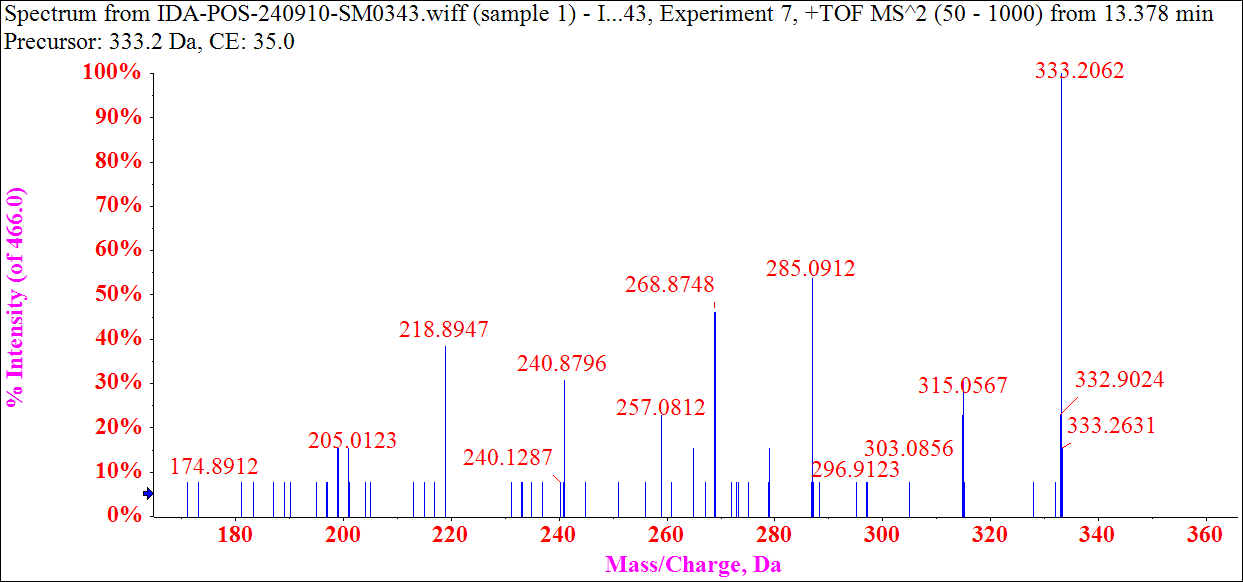 | 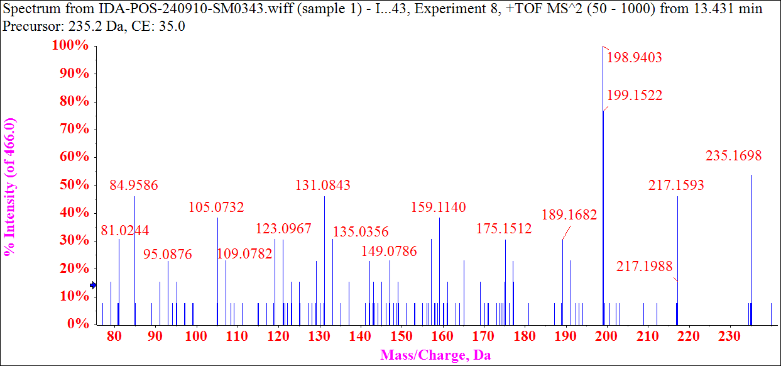  Isopetasol |
| --- | --- |
| 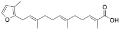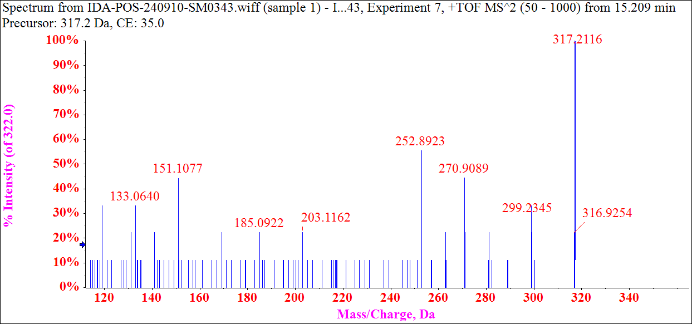  Saurufuran B | 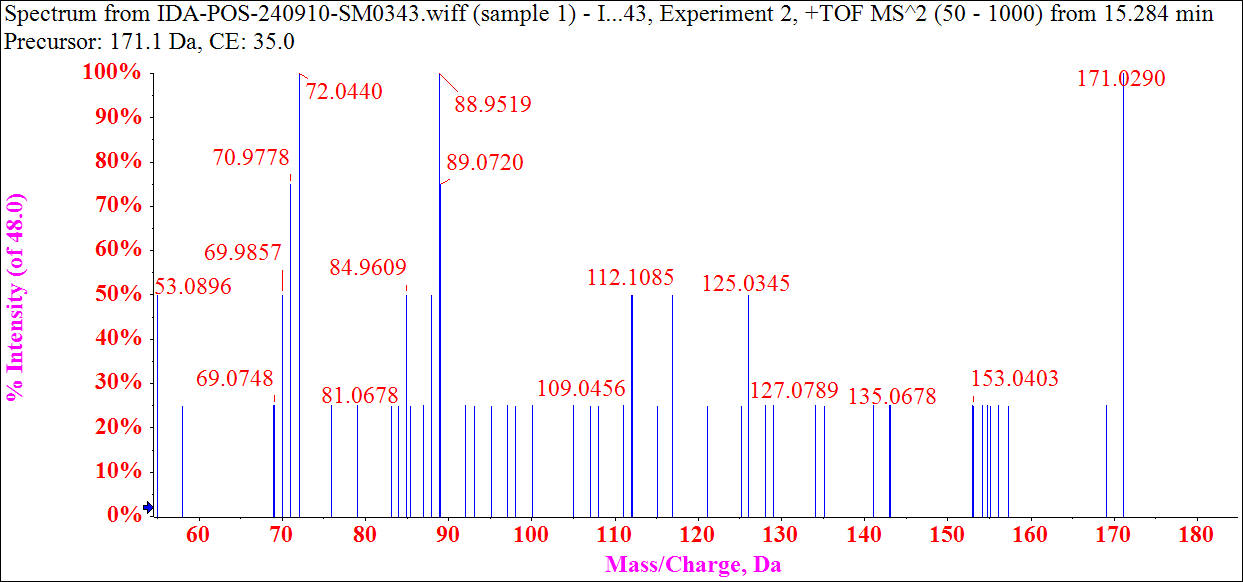 |

| 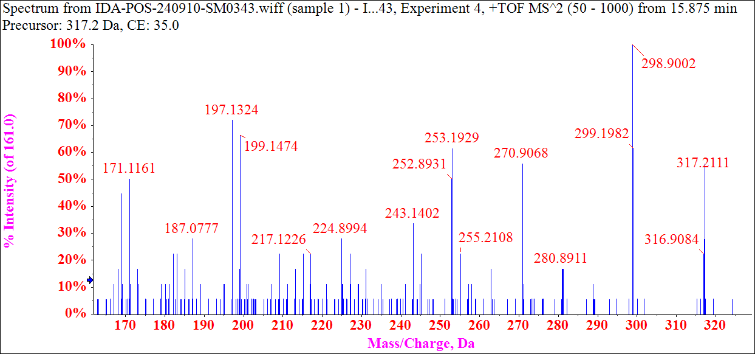 | 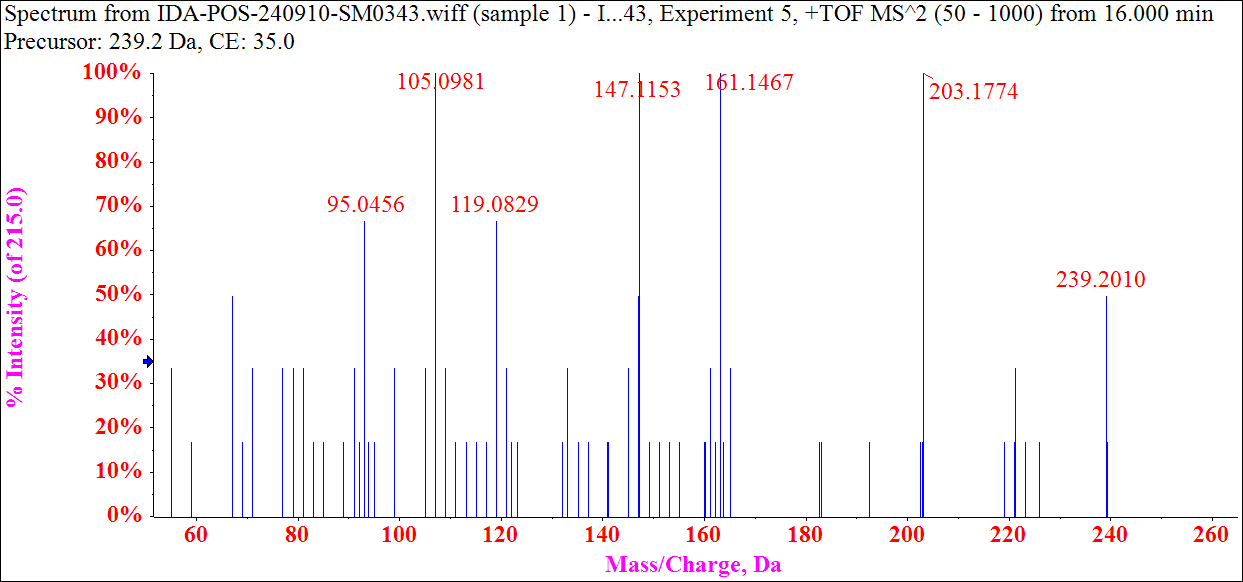 |
| --- | --- |
|  |  |
| 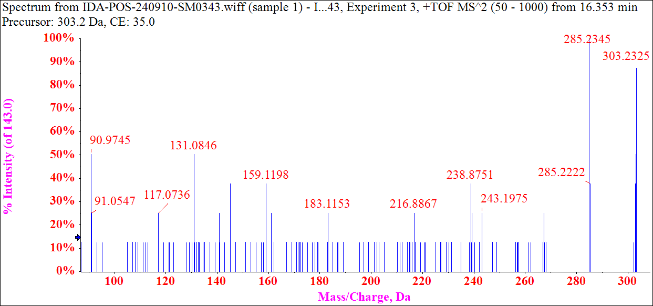 | 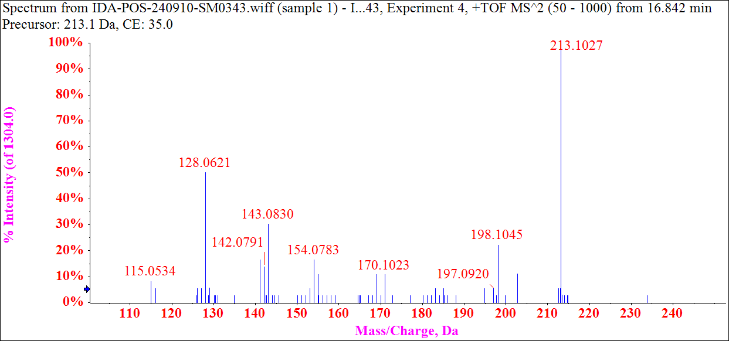 |

| 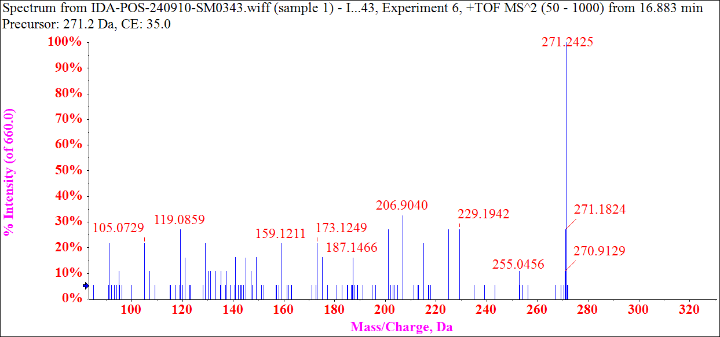 | 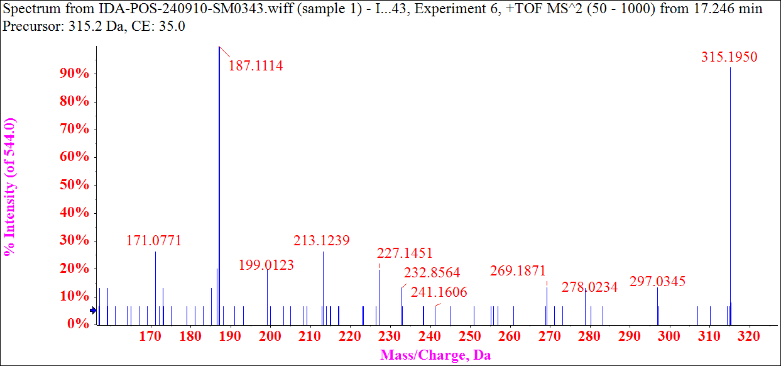 |
| --- | --- |
| 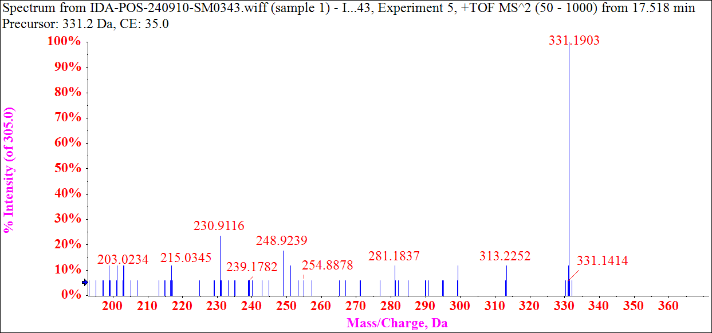 | 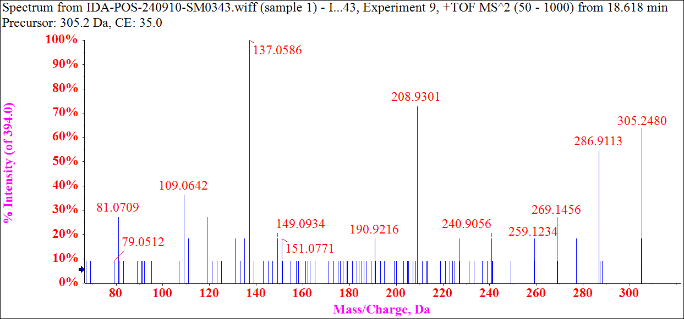 |

| 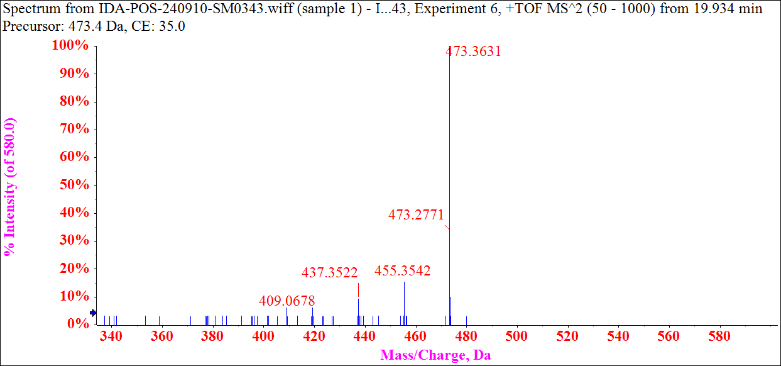 | 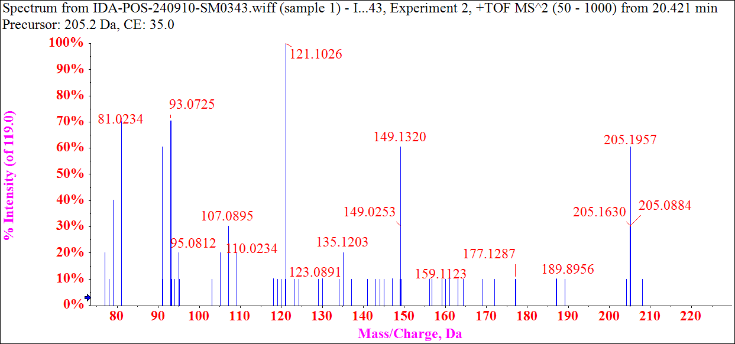 |
| --- | --- |

**Figure S2**. Fragmentation figures of the metabolites identified tentatively in soft coral *L. pauciflorum* by UHPLC-QTOF-MS/MS (positive mode).
